# Simultaneous enzyme production, Levan-type FOS synthesis and sugar by-products elimination using a recombinant *Pichia pastoris* strain expressing a levansucrase-endolevanase fusion enzyme

**DOI:** 10.1186/s12934-022-02009-7

**Published:** 2023-01-26

**Authors:** Ángela Ávila-Fernández, Silvia Montiel, María Elena Rodríguez-Alegría, Luis Caspeta, Agustín López Munguía

**Affiliations:** 1 Centro de Investigación, DACS-Universidad Juárez Autónoma de Tabasco, Av. Gregorio Méndez No. 2838-A. Col. Tamulte ´, 86150 Villahermosa, Centro, Tabasco Mexico; 2 Departamento de Ingeniería Celular Y Biocatálisis, Instituto de Biotecnología, UNAM, Av. Universidad, 2001 Chamilpa, Cuernavaca, Mor. Mexico

**Keywords:** Levan-FOS, Fructans, Prebiotics, *Pichia pastoris*, Levansucrase, Endolevanase

## Abstract

**Background:**

Although Levan-type fructooligosaccharides (L-FOS) have been shown to exhibit prebiotic properties, no efficient methods for their large-scale production have been proposed. One alternative relies on the simultaneous levan synthesis from sucrose, followed by endolevanase hydrolysis. For this purpose, several options have been described, particularly through the synthesis of the corresponding enzymes in recombinant *Escherichia coli.* Major drawbacks still consist in the requirement of GRAS microorganisms for enzyme production, but mainly, the elimination of glucose and fructose, the reaction by-products.

**Results:**

The expression of a fusion enzyme between *Bacillus licheniformis* endolevanase (LevB1) and *B. subtilis* levansucrase (SacB) in *Pichia pastoris* cultures, coupled with the simultaneous synthesis of L-FOS from sucrose and the elimination of the residual monosaccharides, in a single one-pot process was developed. The proof of concept at 250 mL flask-level, resulted in 8.62 g of monosaccharide-free L-FOS and 12.83 gDCW of biomass, after 3 successive sucrose additions (30 g in total), that is a 28.7% yield (w L-FOS/w sucrose) over a period of 288 h.

At a 1.5 L bioreactor-level, growth considerably increased and, after 59 h and two sucrose additions, 72.9 g of monosaccharide-free L-FOS and 22.77 gDCW of biomass were obtained from a total of 160 g of sucrose fed, corresponding to a 45.5% yield (w L-FOS/w sucrose), 1.6 higher than the flask system. The L-FOS obtained at flask-level had a DP lower than 20 fructose units, while at bioreactor-level smaller oligosaccharides were obtained, with a DP lower than 10, as a consequence of the lower endolevanase activity in the flask-level.

**Conclusion:**

We demonstrate here in a novel system, that *P. pastoris* cultures can simultaneously be used as comprehensive system to produce the enzyme and the enzymatic L-FOS synthesis with growth sustained by sucrose by-products. This system may be now the center of an optimization strategy for an efficient production of glucose and fructose free L-FOS, to make them available for their application as prebiotics. Besides, *P. pastoris* biomass also constitutes an interesting source of unicellular protein.

## Background

Fructans are fructose polymers which include inulin and levan, the most abundant structures synthesized by plants or microorganisms respectively. Inulin-type fructooligosaccharides (I-FOS) bearing β2-1 linkages have been recognized as one of the most effective prebiotics due to their demonstrated beneficial health effects in various physiological functions of the human gut microbiota [1]. These I-FOS are commercially available either by enzymatic hydrolysis of inulin extracted from *Chicorium intybus* or by enzymatic synthesis from sucrose by fungal fructosyltransferases [2] and have become common ingredients of functional foods [3, 4]. More recently, it has become clear that alternative fructooligosaccharides, such as levan-type FOS or fructans extracted from agave despite their β2-6 linkages or eventual branching, also bear interesting prebiotic properties [5, 6]. In effect, it is now clear that levan-type FOS, may be used selectively as carbon and energy sources by the beneficial intestinal microbiota [7, 8]. However, unlike I-FOS, which can be produced by a fungal fructosyltransferase (FTF) from sucrose, no FTF-type of enzyme able of synthesizing L-FOS directly has been reported. The closest approach to L-FOS synthesis has been the specific synthesis of low molecular weight (LMW) levan by levansucrases under controlled reaction conditions, as reported by Santos-Moriano et al. [9]. Efforts directed to modify the specificity of levansucrases by site-directed mutagenesis to produce L-FOS have been also carried out without much success [10]. An alternative synthesis of L-FOS consists in a process analogous to chicory I-FOS production [11] where microbial levan is hydrolyzed using an endo-type levanase. Porras-Domínguez et al. (2014) reported a sequential process to produce L-FOS that first involved the synthesis of LMW levan from sucrose using SacB, the levansucrase from *B. subtilis* in *E. coli* as host*,* which once recovered by precipitation with ethanol was subsequently hydrolyzed using LevB1, the endolevanase from *B. licheniformis* [8]. The L-FOS synthesized were finally recovered in the reaction medium. In order to improve the process, avoiding the step of levan recovery after its synthesis, the same authors studied the possibility of carrying out both enzymatic reactions simultaneously. This resulted in a bi-enzymatic synthesis of L-FOS from sucrose with a DP lesser than 10, with levanobiose and blastose as the main products when an equimolar concentration (1 µM) of both enzymes were used in the reaction [12]. The process was further improved through the design of a new biocatalyst fusing both enzymes (LevB1SacB). This option was advantageous considering that the two enzymatic reactions could be handled as one, with a single biocatalyst to produce the L-FOS, bearing the same properties and resulting in the same product profile reported for the bi-enzymatic process [12]. After purification, the L-FOS were assayed in a digestive track simulator reactor with interesting results in terms of the regulation of a microbiota taken from patients suffering from overweight (data not shown).

Nevertheless, a major drawback of any enzymatic L-FOS synthesis with FTFs using sucrose as fructosyl donor is the requirement to eliminate residual glucose, a by-product after the sucrose fructosyl unit is transferred to increase L-FOS or levan chain length. Glucose is also produced as by-product from sucrose hydrolysis, also catalyzed by FTFs. For this purpose, several strategies have been proposed, including the formation of glucose complexes with boronic acids to facilitate its separation [13]. On the other hand, *E. coli* is not a GRAS organism, so the application of products of recombinant enzymes may be limited in the food or pharmaceutical industry or may require complex regulatory procedures. In this context *Pichia pastoris* (*Komagataella phaffii*) could be an alternative to solve both issues: the expression of enzymes in a GRAS microorganism, and the elimination of glucose through its selective consumption (elimination) by the host (*P. pastoris*) in the same system where it is being produced by levansucrase.

*P. pastoris* is an excellent host for recombinant proteins production due, among other reasons, to the availability of strong promoters, both constitutive and inducible [14–16]: to its ability to secrete processed proteins [14, 17, 18]; to its strong preference for respiratory growth, a key physiological trait that greatly facilitates high cell density cultures [15, 16]; to the low levels of endogenous protein secretion into the culture medium [16, 19]; and its GRAS status [14, 17, 18]. Finally, *P. pastoris* does not have invertase activity, making this system idoneous for sucrose transformations.

**Fig. 1:**
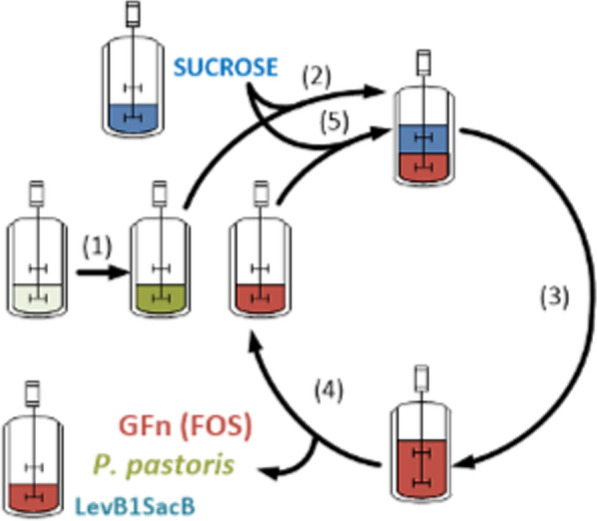
Layout of the simultaneous enzyme production, enzymatic reaction, and glucose elimination fermentation system. (1) Glucose induced Enzyme production (EP) stage; (2) First enzyme production (EP) and enzyme reaction (ER) stages; (3,4,5) Productive fed batch (FB) stages: the enzyme is now induced by residual glucose from the reaction. Half of the culture is retired after each batch and replenished with fresh culture medium containing sucrose. See text for a detailed description

Based on this background, the objective of this work was to synthesize L-FOS from sucrose through a simultaneous bioprocess based on the yeast *P. pastoris* which served as both, the host for recombinant protein expression and as a cellular purification system consuming the free monosaccharides (glucose and fructose), that result from the enzymatic reaction catalyzed by LevB1SacB. We demonstrate here, both at flask and bioreactor level and through several batches of sucrose addition that *P. pastoris* can produce the active fusion enzyme LevB1SacB, which in turn synthesizes L-FOS from sucrose, while the yeast consumes the monosaccharides generated during the reaction as carbon source for growth. All these processes are carried out in a single reactor as illustrated in Fig. 1.

## Results and discussion

### Expression and selection of transformants expressing the fusion enzyme LevB1SacB

The plasmids pPICZαA- LevB_1_SacB and pGAPZαA- LevB_1_SacB, under the control of the methanol inducible pAOX1 promoter and the constitutive pGAP promoter respectively, were used to transform the *P. pastoris* X-33 strain. Five transformants of both X-33/pPICZαA-LevB_1_SacB and X-33/pGAPZαA-LevB_1_SacB were selected in YPDS + Zeocin. The five transformants with the inducible system resulted in the Mut + phenotype, according to their ability to metabolize methanol. The transformants were analyzed in terms of their kinetic performance in 120 h cultures, and the amount of LevB_1_SacB enzyme evaluated (data not shown). The five transformants of the inducible system X-33/pPICZαA- LevB_1_SacB, reached an OD_600_ between 20 and 21 while the pH decreased from 6 to 5.5. Although the activity was detected after 24 h of culture, 776 and 117 U/L for two of the most active transformants, levansucrase activity reached 12,890 and 23,820 U/L of activity after 120 h, with a total protein content of 208–405 mg/L of total protein, respectively.

As far as the constitutive system transformants is concerned (X-33/pGAPZαA-LevB_1_SacB), growth was similar for the five transformants which reached an OD_600_ between 22 and 24 after 120 h when growth had already reached the stationary phase and the pH decreased from 6 to 5.5. The enzymatic activity was quantified after 48 h of fermentation when glucose was exhausted to avoid overestimation of the enzyme activity. Unlike the inducible system, all the constitutive transformants showed a similar behavior, reaching maximums between 2700 and 3076 U/L of levansucrase activity and between 122 and 131 mg/L of protein at 120 h of culture.

The transformants with the highest levansucrase activity of both the inducible and constitutive systems were selected to evaluate the simultaneous expression of enzymes, enzymatic reaction and glucose remotion concept for L-FOS production in fed-batch. Although higher activities were obtained with the inducible strain, a constitutive transformant was selected as it is more appropriate for the designed fed-batch system, avoiding also the need for methanol in the culture.

### Endolevanase activity of LevB1SacB inducible and constitutive

The efficiency of the simultaneous enzymatic reaction (synthesis/hydrolysis) for the synthesis of L-FOS from sucrose, particularly the reaction time for a given L-FOS profile, depends on the levansucrase/endolevanase ratio used [12]. Therefore, the ratio of the levansucrase/endolevanase activities of LevB_1_SacB of the fusion enzyme produced by *P. pastoris* throughout the culture was evaluated.

It was found that the ratio of levansucrase/endolevanase activities of LevB_1_SacB produced in both heterologous expression systems with *P. pastoris* as host were much higher than the ratio observed when LevB_1_SacB was produced in *E. coli* (1.39 U of levansucrase/ U of endolevanase). Considering that both enzymes are part of the same protein, these unexpected large ratio, resulting from a low endolevanase activity, maybe either due to the effect of pH fluctuations on endolevanase stability during the prolonged fermentation time, or to biochemical factors derived from the new host, such as protein glycosylation, partial proteolytic degradation, or incorrect folding of the enzyme, among others [20, 21], affecting exclusively the N-terminal region of the three domain fusion construction. However, as shown by zymography, LevB_1_SacB bears an important endolevanase activity as also measured in the purified LevB_1_SacB forms, so that an additional explanation may be the eventual effect of compounds in the culture medium (methanol or an extracellular metabolite) inhibiting the enzyme or affecting the activity assay.

**Fig. 2:**
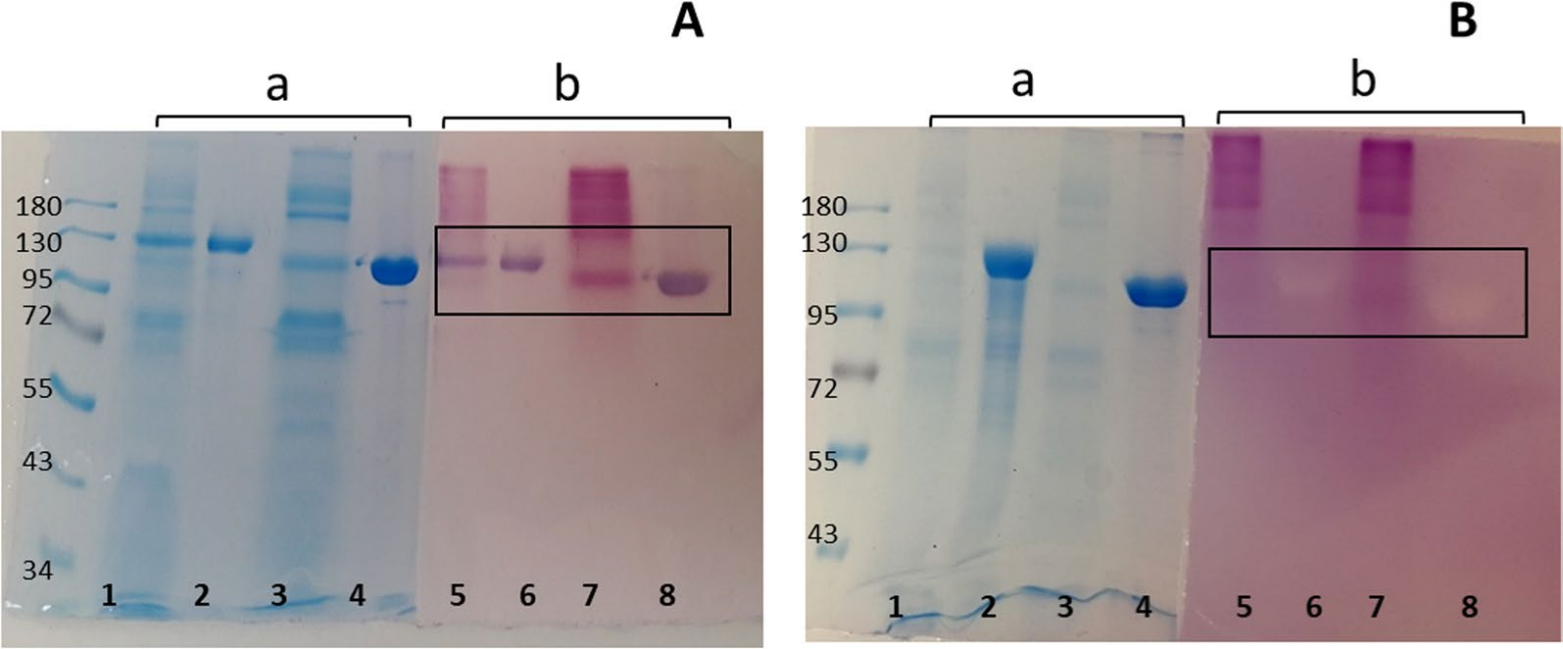
LevB_1_SacB activities: levansucrase (**A**) and endolevanase (**B**). **a** SDS-PAGE and **b** zymography. *Lines 1 and 5*, proteins in the *P. pastoris* X-33/pGAPZαA-LevB_1_SacB culture supernatant; *lines 2 and 6*, LevB_1_SacB purified from the culture supernatant; *lines 3 and 7*, control culture supernatant of *P. pastoris* with X-33/pGAPZαA without the *LevB*_*1*_*SacB* gene; *lines 4 and 8*, purified LevB_1_SacB expressed in *E. coli* culture

In Fig. 2, levansucrase activity zymograms from constitutive LevB_1_SacB in *P. pastoris* compared to LevB_1_SacB produced in *E. coli* are shown. A band demonstrating the levansucrase enzymatic activity with a molecular weight corresponding to a 130 kDa protein was clearly observed, both in the culture medium, as well as in the purified protein solutions obtained from the constitutive *P. pastoris* culture media. Interestingly, the equivalent molecular weight of the band (around 130 kDa) was slightly higher than that of the fusion enzyme expressed in *E. coli* Rosetta 2. This difference in molecular weight is a common feature when expressing proteins in *P. pastoris* [19, 22], and a probable explanation to a endolevanase behavior.

As already mentioned, the endolevanase activity assay measured in the LevB_1_SacB fusion protein produced in *P. pastoris* was lower than the activity expected when compared to the activity of both enzymes in the LevB_1_SacB construction expressed in *E. coli*. However, the zymography obtained from the same solutions showed that the endolevanase activity of both LevB_1_SacB produced in *E. coli* and LevB_1_SacB produced in the constitutive *P. pastoris* strain were comparable, as also shown in Fig. 2.

### Design of a simultaneous enzyme production, enzymatic reaction, and glucose elimination system

Once we were able to demonstrate that LevB_1_SacB produced in the constitutive *P. pastoris* culture system possesses both enzymatic activities and behaves similarly than the enzyme from *E. coli* [12] a process scheme was designed to take advantage of several physiological properties of the *P. pastoris* culture for the synthesis of L-FOS from sucrose as the following:

- *Enzyme production (EP)*. The enzyme is produced constitutively with the pGAP promoter without requirement of methanol induction as in the case of the inducible system, which in spite of the higher amount of enzyme produced could limit further applications of the culture product as food supplement or would impose intensive purification.
- *Enzyme reaction (ER)*. Enzyme reaction will take place by addition of sucrose as substrate to the culture medium where the enzyme is constitutively produced.
- *Batch Culture (BC). P. pastoris* will continue growing at the expense of glucose (and eventually fructose), by-products of the enzymatic reaction. More enzyme will be synthesized by *P. pastoris*. Neither sucrose nor the synthesized L-FOS are consumed by the yeast, so that the former will be exclusively used as substrate by LevB_1_SacB, while the product, L-FOS, will accumulate in the supernatant.
- *Fed batch operation (FB).* Sucrose is provided in a fed-batch mode together with the medium components to allow for further growth, enzyme production associated to growth and L-FOS synthesis with the concomitant liberation of glucose, as long as sufficient enzyme is available. After each sucrose and medium addition, the same amount of medium containing the accumulated L-FOS may be simultaneously withdrawn as product.

Considering that experiments were performed initially at Erlenmeyer and later at bioreactor level of a defined volume (Vf), the following is a general layout of the production system also illustrated in Fig. 1:

1. *EP stage*. Culture of *P. pastoris* X-33 in glucose for the constitutive production of LevB_1_SacB in an initial fermentation volume of 0.5Vf.
2. *EP and ER stages*. After 72 h of culture in glucose, addition of 0.5Vf L of fresh culture medium containing 200 g/L of sucrose, to reach a volume of Vf L containing 100 g/L of sucrose. *P. pastoris* will continue to grow, at this stage at the expense of the by-products of L-FOS synthesis by LevB_1_SacB from sucrose. Associated to *P. pastoris* growth, the constitutive production of the fusion enzyme will continue.
3. *Productive FB stages.* As sucrose is depleted, the stepwise withdrawal of 0.5Vf L from the culture medium proceeds with the concomitant addition of the same volume (0.5Vf L) of fresh culture medium containing 200 g/L of sucrose. The number of production stages can be as large as the process allows. Theoretically, at the end of each productive stage, the 0.5Vf L withdrawn containing biomass, LevB1SacB, and the synthesized product (L-FOS) without glucose, fructose or residual sucrose are collected as product.

As observed, the proposed process is an innovative simultaneous enzyme production, enzymatic reaction and glucose elimination system, also providing *P. pastoris* unicellular biomass protein as an interesting product.

### Process evaluation at flask-level

**Fig. 3:**
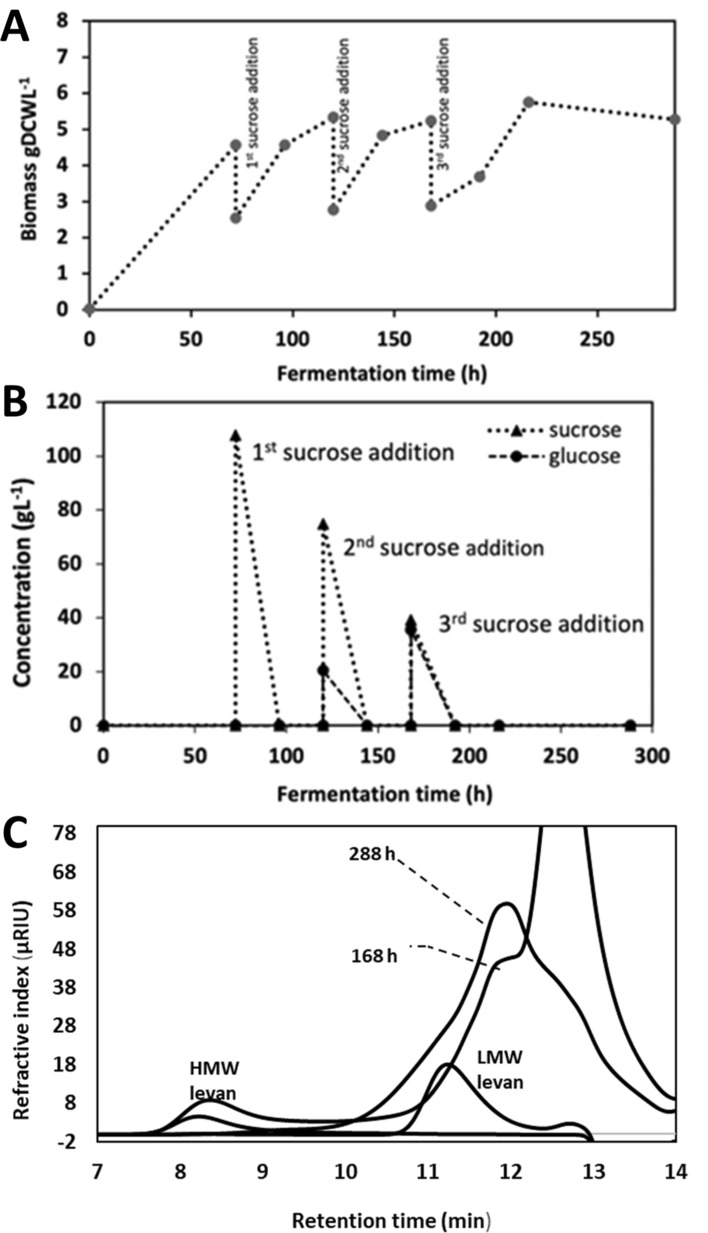
**A** Growth kinetics of recombinant *P. pastoris* X-33/pGAPZαA- LevB_1_SacB system at flask-level on BMY medium with three sucrose additions at 72, 120 and 168 h. Reaction conditions: 28 °C, 200 rpm, final volume of 0.1 L and total sucrose concentration of 300 g/L supplied in three stages. **B** Sucrose and monosaccharides consumption along the culture. **C** GPC of levan molecular weight distribution of the final products compared to the 3rd sucrose addition

Figure 3a shows that at the Erlenmeyer flask level, growth was associated to glucose consumption during the first 72 h of culture, reaching 4.57 gDCW/L of biomass. At this point, the limiting substrate (glucose) had been consumed (Fig. 3b) and 4100 U/L of LevB1SacB (quantified as levansucrase activity) had been produced constitutively and secreted to the culture medium. The yeast resumes growth after each addition of sucrose in a fresh culture medium, using the free monosaccharides that result from the enzymatic reaction. Interestingly, similar amounts of biomass were obtained at the end of each stage, that is 5.35, 5.23 and 5.27 gDCW/L of biomass collected at 120, 168 and 288 h, respectively. Along this process, the culture pH decreased from 6 to 4 a consequence of the organic acids produced probably due to the increased oxygen limitation as biomass increased, compared to the first hours of fermentation.

Figure 3b also shows that in the L-FOS production stages, the sucrose fed dropped to lower values than the theoretical 100 g/L, probably due to the high levansucrase activity. This is confirmed by the simultaneous increase in glucose as a by-product of both the transferase and the hydrolytic SacB reactions. Surprisingly, after each step of the batch cultures, all sucrose added with the fresh culture medium was consumed, reaching 100% conversion after 24 h of culture. Even more interesting was the fact that no free fructose was detected in the reaction medium, implying either a minimum enzymatic hydrolysis of sucrose by SacB during the process, and/or that fructose was consumed as fast as glucose as carbon source by *P. pastoris.* An additional possibility was that fructose is used by SacB in the fusion enzyme as a fructose acceptor to produce levanbiose and from this acceptor, oligo-levans eventually.

**Fig. 4:**
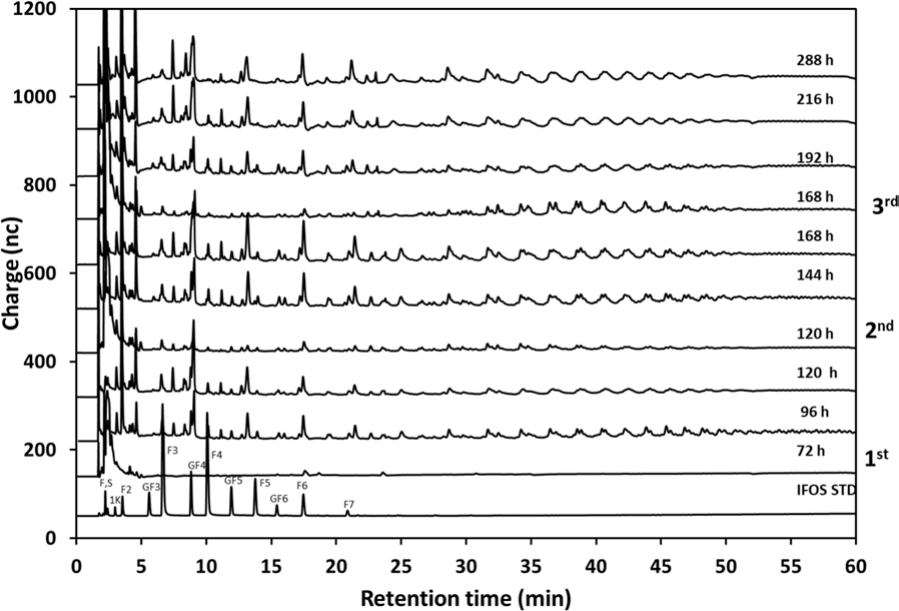
HPAEC-PAD. Profile of L-FOS produced by the system at flask-level. The process was developed for three sucrose additions (100 g/L) 1st at 72, 2nd at 129, and 3rd at 168 h, 28 °C and 200 rpm in a volume of reaction of 0.1 L. F:fructose, S:sucrose, 1 K: 1-kestose, F2:inulobiose, GF3:nistose, F3:inulotriose, GF4:fructosyl-nistose, F4:inulotetraose, GF5:kestohexaose, F:inulopentaose, GF6:kestoheptaose, F6:inulohexaose y F7: inuloheptaose

The kinetics of products formation, particularly the L-FOS profile, were evaluated throughout the process under two different perspectives. One was the possibility of accumulation of SacB synthesis products (levan of either low or high molecular weight distribution), a consequence of insufficient endolevanase activity. Through GPC analysis of the products from samples collected after each batch, we found that HMW levan was hydrolyzed by endolevanase only at the end of the process (Fig. 3c), while no LMW levan accumulated indicating its rapid hydrolysis after synthesis. These results confirmed the higher affinity of endolevanase reported for LevB1SacB towards small levan polymers, preventing their accumulation [8]. The second perspective involves directly the dynamics of the L-FOS profile obtained throughout the process. We found that 24 h after the first sucrose addition, L-FOS with a maximum DP of 20 were obtained and the profile remained unchanged all throughout the process until the last batch explored (Fig. 4). This may be the consequence of a process controlled by yeast growth leaving enough time for the parallel process of L-FOS synthesis by the secreted enzyme. The main product of the system (an F2) was identified from a commercial I-FOS standard (Raftylose P95 from Orafti) as levanbiose, as also found in the batch synthesis of L-FOS using the simultaneous reaction with the biocatalyst LevB1SacB produced in *E. coli* [12]. Levanbiose results mainly from the direct hydrolysis by endolevanase from the levan chains, as demonstrated by Porras-Domínguez et al. in kinetic experiments analyzed by TLC [8]. The presence of larger L-FOS molecules was clearly defined in the chromatogram, mainly F3, F4, F5 and F6 and to a lesser extent F7, F8, F9 and so on up to F20. Finally, the small signals that appear between each of the products of this series, known as the Fn series, could correspond to FOS of the 1-kestose, 6-kestose, blastose series and those including branches in β 2–1 [23].

At the end of the process, 8.62 g of total L-FOS were obtained from 30 g of sucrose added during the 3 stages, which was equivalent to a yield of 28.7 g of L-FOS per 100 g of sucrose, free of monosaccharides and residual sucrose. In Table 1 the batch and fed batch L-FOS production processes in flask and bioreactor systems are reported.

**Table 1:** Comparison of the reaction medium composition at the end of the Fed batch flask and the bioreactor *P. pastoris* cultures expressing LevB_1_SacB under the GAP promoter, and the reaction using LevB_1_SacB produced by *E. coli*

	Time (h)	L-FOS/Sucrose (g/ g)	Glucose (g)	Fructose (g)	Residual sucrose (g)	Maximum L-FOS DP	Total *P. pastoris* biomass (g)
X-33/pGAPZαA-LevB_1_SacB (Flask-level)	288	0.28	0	0	0	20	1.05
X-33/pGAPZαA-LevB_1_SacB (Bioreactor scale)	59	0.45	0	0	6.83	10	21.85
Rosetta 2/PET-22 ( +) LevB_1_SacB *	6	0.41	54 g/L	234 g/L	65 g/L	10	–

Data taken from Porras-Domínguez, 2017

### Evaluation of the process at bioreactor scale

In order to scale up the process, and as a proof of concept, the simultaneous fermentation/reaction process was carried out in a 1.5 L stirred bioreactor, with a 0.8 L of final working volume (Vf). A similar procedure and culture conditions already reported for the flask-level experiments were maintained, as well as the culture feeding and removal strategy. Unlike the flask process, the fermenter allowed for the control of pH and dissolved oxygen.

**Fig. 5:**
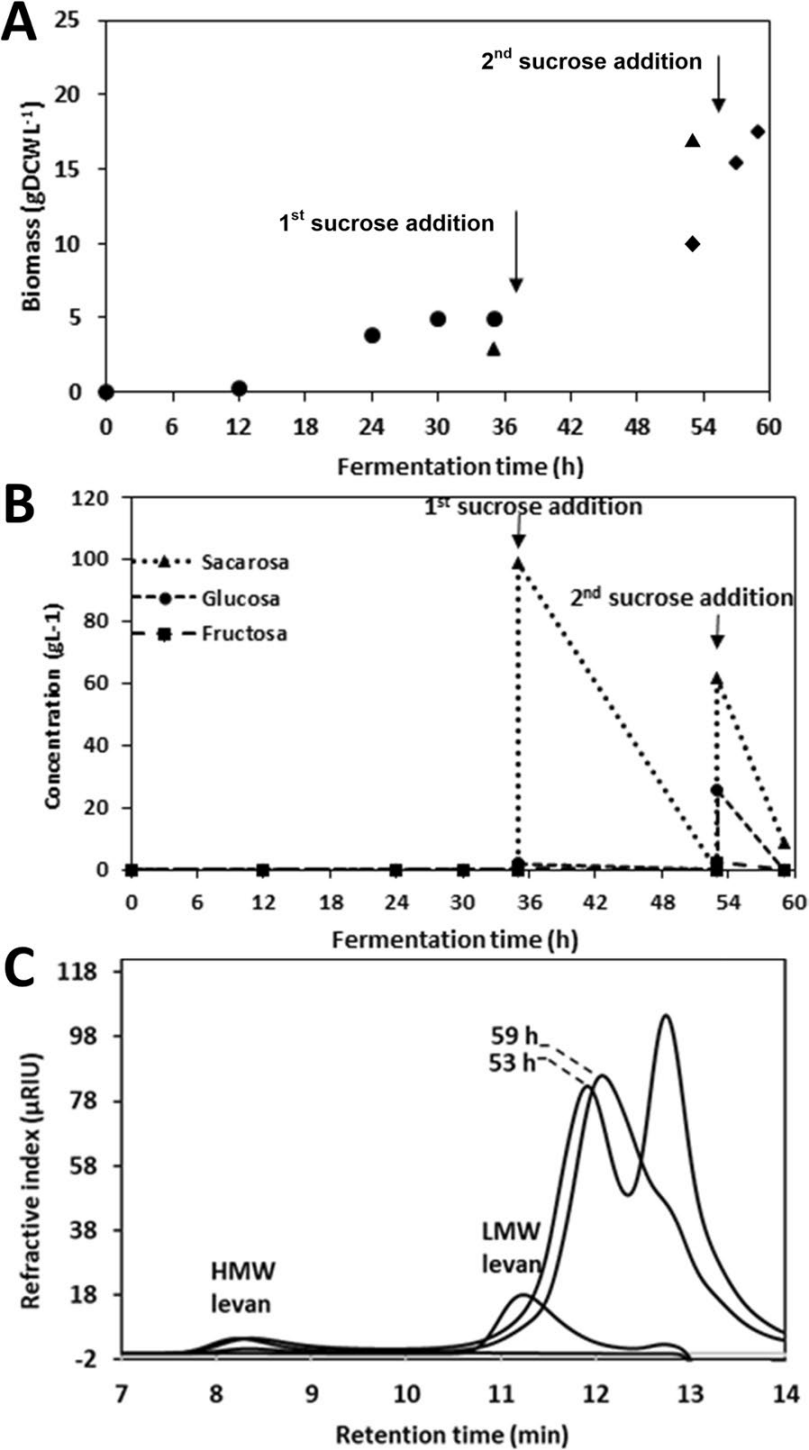
**A** Growth kinetics of recombinant *P. pastoris* X-33/pGAPZαA-LevB1SacB system. Bioreactor scale on BMY medium with two sucrose additions at 35 and 53 h. Reaction conditions: 28 °C, 200–1200 rpm, final volumen of 0.8 L and total sucrose concentration of 200 g/L supplied in two stages. **B** Sucrose and monosaccharides consumption along the culture. **C** GPC. Levan molecular weigth distribution of the final products compared to the 2nd sucrose addition

Figure 5a shows that *P. pastoris* growth reached 4.95 gDCW/L in the initial culture stage. Subsequently, when growth was based on monosaccharides as carbon an energy sources, biomass increased to 17.05 and 17.48 gDCW/L, in the next two stages after 18 and 6 h respectively. These results clearly show that the fermenter was more efficient in terms of biomass production than shake-flasks cultures due to the extent of oxygen availability. In the same way, pH control favors the stability of the enzymes in the fusion peptide, allowing for a better operation in optimal pH conditions. As far as the enzymatic reaction is concerned, the volumetric activity of the synthesized fusion enzyme, increased throughout the fermentation time, reaching a total of 15,120 U/L measured as levansucrase activity at the end of the process. Figure 5b shows that sucrose was completely consumed after 18 h of the first sucrose addition, while no glucose or fructose were detected in the medium. Similarly, as in the flask assay, we observed that in the second stage, sucrose was rapidly consumed after addition, releasing glucose into the reaction medium. After 59 h of the total process a certain amount of residual sucrose was still present, but as in the previous experiments in erlenmeyer, all monosaccharides had been consumed (Table 1). In the same Table a comparison is made with the previous *E.coli* batch process, where residual sugars are present. Considering that the enzyme remained active in the collected medium, all sucrose is eventually consumed. Therefore, the final L-FOS profile may be defined both, by the preferential final products of the endolevanase and the last stage reaction time.

**Fig. 6:**
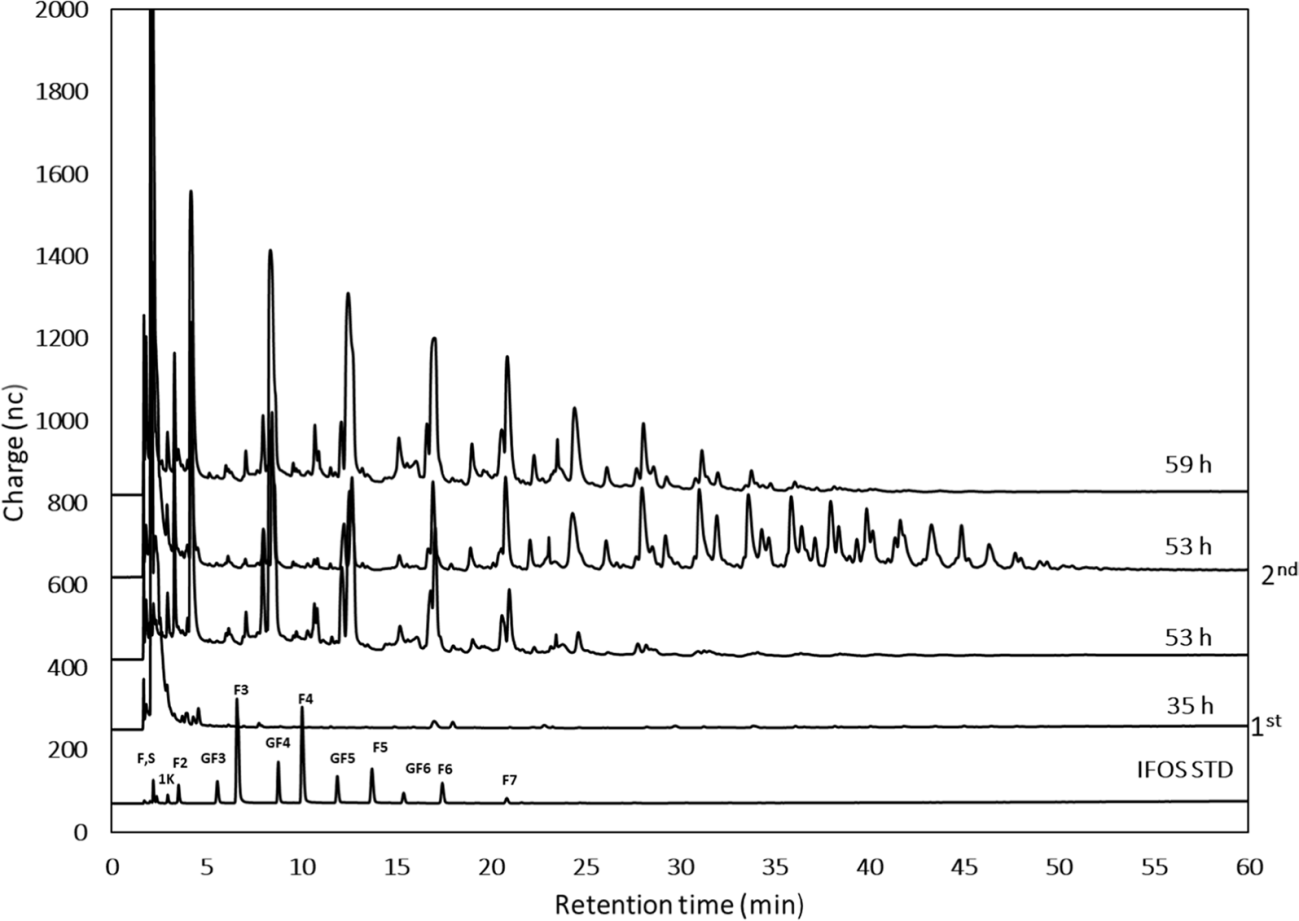
HPAEC-PAD. Profile of L-FOS produced by the system at bioreactor scale. The process was run with two sucrose additions (100 g/L) 1st at 35 and the 2nd at 53 h, 28 °C and 200–1200 rpm in a final volume of reaction of 0.8 L. F:fructose, S:sucrose, 1 K: 1-kestose, F2:inulobiose, GF3:nistose, F3:inulotriose, GF4:fructosyl-nistose, F4:inulotetraose, GF5:kestohexaose, F:inulopentaose, GF6:kestoheptaose, F6:inulohexaose y F7: inuloheptaose

It was also observed that HMW levan polymer and large DP L-FOS did not accumulate at the end of the process (Fig. 5c), as a consequence of enough endolevanase activity to deal with the levan synthesized by levansucrase. As far as the L-FOS profile is concerned, products with a maximum DP of 8 are observed after 18 h of reaction following the first addition of sucrose (Fig. 6). These are mainly L-FOS of the Fn series, that is, those resulting from the endo-type hydrolysis on levan, without a glucose molecule in one end of the chain. Subsequently, immediately after the second sucrose addition, the synthesis of higher DP L-FOS, associated to high levansucrase activity, are observed. At this point, the accumulated volumetric levansucrase activity reached 11.42 U/mL, 5 times higher than the activity measured when the reaction started (2.22 U of levansucrase activity/mL). Interestingly, at the end of the second stage, the highest L-FOS DP was 10, with a product profile different from the one observed in the first stage, as a result of the lower time available for the reaction leading to a less extensive levan hydrolysis. These results demonstrate the complex dynamics of this reaction system, particularly in terms of the L-FOS final profile, which depends on the endolevanase specificity, as well as on reaction time and exposure to endolevanase after collection. When the reaction time is limited, as in the previous stages, even when sucrose is no longer available, intermediate hydrolysis products are observed.

Finally, at the end of the 59 h process developed at a bioreactor level an accumulation of 72.9 g of L-FOS were obtained from 160 g of sucrose fed in two stages. This is equivalent to a global yield of 45.5 g of L-FOS per 100 g of sucrose, a 1.6 times higher yield than the yield obtained in flask level. However, the novelty of this process relies on the fact that the products are obtained in a glucose and fructose free medium. In terms of monosaccharide-free L-FOS production, at the bioreactor level, the process behaved similarly as in the Erlenmeyer flask experiments, as shown in Table 1. However at the bioreactor level, another major advantage is that *P. pastoris* reaches a higher cell density since it is possible to simultaneously monitor and control pH and dissolved oxygen [15, 16, 19, 22]. At the fermenter level, we obtained a higher efficiency for the constitutive production of SacBLevB, the fusion enzyme, as the concentration of secreted heterologous proteins is proportional to cell concentration [15, 16]. The amount of secreted and accumulated enzyme is essential for a successful conduction of the project, as it is critical in one hand to provide sugars from the reactions for *P. pastoris* growth, and in the other hand, to eliminate them, which is one of the main objectives of the system. The glucose synthesis/consumption rates should be adequately balanced to avoid growth limitation by the carbon source or excessive accumulation.

Another advantage of conducting the process in a fermenter relies on the higher productivity of L-FOS, as the synthesis occurs almost 5 times faster than in shaken flask and with an almost double production yield, equivalent to 45.5 vs 28.7 g of L-FOS/ 100 g of sucrose. Namely, L-FOS were produced at 0.26 g/L/h in flasks, compared to 1.24 g/L/h in bioreactor.

The main advantage of this novel *P. pastoris* fermentation/reaction system for the synthesis of L-FOS over the previously reported process where the gene coding for the fusion protein biocatalyst was expressed in *E. coli,* is that L-FOS are obtained free from glucose and fructose, while the sugars must be removed in the process based in the *E. coli* recombinant enzyme [12]. In addition, the continuous elimination of glucose and fructose allow a reaction without product inhibition, so that total sucrose conversion is rapidly reached, as summarized in the results presented in Table 1. It is also worth mentioning that although the fusion biocatalyst produced in *E. coli* has a higher endolevanase activity compared to *P. pastoris* as previously discussed, the endolevanase in the *P. pastoris* biocatalysts, is enough to assure the continuous hydrolysis of levan, even after collection of the culture and until enzyme deactivation for further processing. This was the case in the *E. coli* biocatalysts which require a limited reaction time to prevent modifications of the final L-FOS profile due to endolevanase hydrolysis. The extension of the reaction time also allows for the hydrolysis of residual high molecular weight levan.

## Conclusions

We have demonstrated here the technical feasibility of a simultaneous system performing a fermentation and an enzymatic reaction, where the growth of a recombinant *P. pastoris* strain results in the synthesis of an enzyme whose activity provides sugars as by-products used as a carbon source in the fermentation. This system allows the production of glucose and fructose free L-FOS with a DP between 2 and 10, with levanobiose (F2) as the most abundant product. The system may be optimized in order to increase its efficiency and define process conditions according to a particular L-FOS profile. Minimum media will be designed and optimized in order to decrease production costs. Nevertheless, the elimination of glucose and fructose in this novel one reactor fermentation/reaction system for the synthesis of L-FOS is an innovative approach for the synthesis of these and other oligosaccharides required in a purified form for application as prebiotics. This could also be the case for gluco-oligosaccharides and galacto-oligosaccharides that could be produced by glucosyltrasferases and beta-galactosidases respectively from disaccharides as substrate donating a glycosyl unit using a similar approach.

## Methods

### Strains and plasmids

The sequence LevB_1_SacB was synthesized with the codon usage optimized to *P. pastoris* by GenScript USA Inc and cloned into the plasmids pPICZαA and pGAPZαA using the EcoRI and XbaI restriction sites. *E. coli* Top 10 (Invitrogen, CA, USA) was used as maintenance strain for the plasmids, while *P. pastoris* X-33 (Invitrogen, CA, USA) was used as the production strain.

#### P. pastoris transformations

*pPICZαA-LevB*_*1*_*SacB* and *pGAPZαA-LevB*_*1*_*SacB* were linearized using the restriction enzymes Sac I and Avr II, respectively. 80 µL of *P. pastoris* X-33 electrocompetent cells were electroporated using the linearized plasmid and recovered in sorbitol 1 M at 28 °C for 2 h without agitation. Transformants were selected in YPDS + Zeocin Agar (Yeast extract 10 g/L, Peptone 20 g/L, Glucose 20 g/L, Sorbitol 182 g/L, Bacto agar 20 g/L and Zeocin 100 µg/mL), cultivated in liquid YPD + Zeocin (Yeast extract 10 g/L, Peptone 20 g/L, Glucose 20 g/L,, and Zeocin 100 µg/mL), cryopreserved with 40% (v/v) glycerol and stored at -70 °C. *pPICZαA* and *pGAPZαA* without the *LevB*_*1*_*SacB* gene were linearized and used to transform *P. pastoris* X-33 electrocompetent cells using the same conditions. The *P. pastoris* X-33/pPICZαA and X-33/pGAPZαA transformants were used as control.

### Expression of the fusion enzyme LevB_1_SacB

A single transformant of *P. pastoris* X-33/pPICZαA-LevB_1_SacB or X-33/pGAPZαA-LevB_1_SacB was inoculated in 3 mL YPD + Zeocin and incubated at 28 °C and 200 rpm for 24 h as pre-inoculum.

*P. pastoris* X-33/pPICZαA-LevB_1_SacB pre-inoculum was used to inoculate 25 mL BMGY (yeast extract 10 g/L, peptone 20 g/L, YNB 13.4 g/L, biotin 4*10^–4^ g/L, 1% glycerol, and pH 6.0, 0.1 M phosphate buffer) at 0.1 OD_600_. The culture was incubated at 28 °C and 200 rpm until an OD_600_ of 2 was reached. Cells were then recovered by centrifugation at room temperature (2500 g × 5 min) and washed twice with 0.1 M, pH 6 phosphate buffer. Cells were resuspended in 50 mL BMMY (yeast extract 10 g/L, peptone 20 g/L, YNB 13.4 g/L, biotin 4*10^–4^ g/L in pH 6, 0.1 M phosphate buffer) with 0.5% methanol as inducer of LevB_1_SacB production in the culture, starting with an OD_600_ of around 1 and replenishing methanol every 24 h for a total of 120 h.

Alternatively, a *P. pastoris* X-33/pGAPZαA-LevB_1_SacB pre-inoculum was used to inoculate 50 mL BMY + Glucose (yeast extract 10 g/L, peptone 20 g/L, YNB 13.4 g/L, biotin 4 × 10^–4^ g/L, 2% glucose and 0.1 M pH 6 phosphate buffer) at 0.1 OD_600_. The culture was incubated at 28 °C and 200 rpm for 120 h.

In both cases, growth (OD_600_), pH and enzymatic activities (levansucrase y endolevanase) were measured daily. *P. pastoris* X-33/pPICZαA or X-33/pGAPZαA transformant cells treated under the same conditons were used as controls.

### Standard levansucrase (SacB) and endolevanase (LevB_1_) activity assays

Levansucrase (SacB) and endolevanase (LevB_1_) initial reaction rates were determined in the fusion enzyme LevB_1_SacB following the initial reducing sugar release by the DNS method [24] from the corresponding substrates as described below, both reactions at 37 °C and 500 rpm in 50 mM pH 6.0. acetate buffer containing 1 mM CaCl_2._ Levansucrase activity was assayed in 600 μl reactions and 100 g/L sucrose as substrate while endolevanase in 500 μl reactions and 10 g/L of 8.3 KDa average molecular weight levan as substrate. Levan was produced enzymatically with *B. subtilis* SacB as described by Porras-Domínguez et al. (2015) [25]. One unit of levansucrase or endolevanase activity (U) was defined as the amount of enzyme releasing 1 μmol of glucose or fructose equivalents per minute, respectively. All activity assays were carried out by duplicates.

### Preotein concentration and zymography

Total and purified proteins from the culture media were measured by the Bio-Rad protein assay using bovine serum albumin (BSA) as standard. 10% SDS-PAGE gels were prepared as described by Laemmli [26]. For the endolevanase activity assay, gels included 1% w/v LMW SacB levan (8.3 kDa) in the formulation. Purified LevB_1_SacB (10 µg) was charged in control gels as well as gels containing levan.. After migration, gels were washed at room temperature three times for 30 min in a refolding buffer (1% v/v Tween 80 in pH 6.0, 50 mM sodium acetate buffer). Finally, gels were incubated in the same buffer overnight, in order to visualize the endolevanase activity in situ*.* An additional set of experiments were performed to visualize levansucrase activity, incubating overnight the levan-free gels in the buffer containing 10% (w/v) sucrose. The use of reducing agents was avoided during SDS-PAGE.

The lanes containing the molecular weight standard were cut off and stained with Gel Blue Stain Reagent (Thermo Scientific), while those containing LevB_1_SacB were stained with the Schiff reagent (Sigma, St. Louis, MO). For this purpose, the gels were incubated for 30 min in a 75% v/v ethanol solution, followed by a 1 h incubation in a periodic (0.7%) and acetic (5%) acids solution. Afterwards, several washing steps were performed with a 0.2% sodium bisulfite and 5% acetic acid solution until the gels became clear. Finally, the gels were placed in contact with the Schiff reagent. LevB1 activity is observed when the whole gel is stained except for the regions where endolevanase activity degraded the levan polymer in the gel, while SacB activity is observed as a single stained band corresponding to the polymer synthesized by the enzyme.

### Recomnbinant ***P. pastoris*** growth, LevB_1_SacB production and L-FOS synthesis in Erlenmeyer flasks

In a first step a single transformant of *P. pastoris* X-33/pGAPZαA-LevB_1_SacB was inoculated in 3 mL YPD + Zeocin and incubated at 28 °C and 200 rpm for 24 h. This pre-inoculum was used to inoculate 50 mL BMY + Glucose at 0.1 OD_600_, incubated in the same conditions for 72 h. Up to this point glucose supported growth and LevB_1_SacB production. Afterwards, a fed batch culture was started when the 50 mL of the culture containing the induced enzyme were complemented with 50 mL of fresh BMY medium but this time containing 200 g/L sucrose to complete 100 mL with a final sucrose concentration of 100 g/L in a 250 mL Erlenmeyer flask and incubated. Twice after 48 h, a volume of 50 mL of culture broth was withdrawn and replenished with 50 mL of fresh medium (BMY + 200 g/L sucrose). After the last withdrawn/replenish procedure, the medium was incubated for an additional period of time (120 h) to allow for sucrose conversion and further cell growth on glucose and fructose, issued from the enzymatic reaction. Therefore L-FOS were produced in three stages, the first two lasting 48 h and the last one 120 h. If the initial 72 h of culture in glucose are considered, then the whole process lasted 288 h.

### ***P. pastoris*** growth, LevB_1_SacB production and L-FOS synthesis in Bioreactor

A single transformant of *P. pastoris* X-33/pGAPZαA-LevB_1_SacB was inoculated in 20 mL YPD + Zeocin and incubated at 28 °C and 200 rpm for 24 h. This culture was then used to inoculate a 1.5 L Applikon bioreactor (Applikon ADI 1010/ ADI 1025, Delft, The Netherlands), containing 400 mL BMY + Glucose at 0.1 OD_600_. In the reactor, the initial culture conditions were 28 °C, 330 rpm with an aeration rate of 0.5 vvm. pH was adjusted by automatic addition of 4 M KOH; the concentration of dissolved oxygen was not allowed to decrease from 40%, increasing the agitation rate up to 1200 rpm. After 35 h the fed batch culture was started with the addition of 400 mL BMY + 200 g/L sucrose to reach 800 mL of total volume in the reactor,  then the culture proceeded for 18 h. Again, 400 mL of culture were withdrawn and replenished with 400 mL of fresh BMY + 200 g/L sucrose for an additional 6 h. During this time, the aeration rate increased to 1.0 vvm. At each stage the biomass and the enzyme activity were measured. If the initial 35 h of culture in glucose are considered, then the whole process lasted 59 h.

### Identification and quantification of carbohydrates

Glucose, fructose, residual sucrose, levan and L-FOS were measured at the different stages of the process. Glucose, fructose and sucrose in the culture medium were quantified in a HPLC system (Ultimate 3000, Dionex) equipped with a refractive index detector (RefractoMax 520) using a Gold Amino (4.6 × 250 mm) column at 30 °C and 1 mL/min acetonitrile:water (75:25) as eluent. Levan was quantified by gel permeation chromatography (GPC) using the same system but equipped with an Ultrahydrogel™ Linear (7.8 × 300 mm) column at 35 °C and using 0.1 M sodium nitrate 0.8 mL/min as eluent. High and low molecular weight SacB Levan produced as described by [25], were used as standard. L-FOS were identified in a HPAEC-PAD system (Dionex) equipped with an electrochemical detector (ED50 Dionex) using a CarboPac PA-200 (2 × 250 mm) column equilibrated at 30 °C with 0.5 mL/min of 0.1 M NaOH. Fructans were eluted with a sodium acetate gradient (5 mM for 5 min, followed by a 5–230 mM linear gradient in 95 min). I-FOS (Raftilosa P95 de Orafti) with DP from 2 to 7 was used as standard to identify some of the products.

## Data Availability

All data are included in the manuscript. Further queries or additional information can be requested to the corresponding author.

## Abbreviations

BC: Batch Culture
DP: Degree of polymerization
EP: Enzyme production
ER: Enzyme reaction
FB: Fed batch operation
FTF: Fungal fructosyltransferase
GPC: Gel Permeation Chromatography
GRAS: Generally recognized as safe
HPAEC-PAD: High-performance anion-exchange chromatography/pulsed amperometric detection
HPLC: High-performance liquid chromatography
I-FOS: Inulin-type fructooligosaccharides
L-FOS: Levan fructooligosaccharides
LevB1: *Bacillus licheniformis* Endolevanase
LevB1SacB: *Bacillus licheniformis* Endolevanase (LevB1) and *Bacillus subtilis* levansucrase (SacB) fusion
LMW: Low molecular weight
OD: Optical density
SacB: *Bacillus subtilis* Levansucrase
SDS-PAGE: Sodium dodecyl-sulfate polyacrylamide gel electrophoresis
